# Modulation of human endogenous retrovirus (HERV) transcription during persistent and *de novo* HIV-1 infection

**DOI:** 10.1186/s12977-015-0156-6

**Published:** 2015-03-24

**Authors:** Michelle Vincendeau, Ingmar Göttesdorfer, Julia M H Schreml, Armand G Ngounou Wetie, Jens Mayer, Alex D Greenwood, Markus Helfer, Susanne Kramer, Wolfgang Seifarth, Kamyar Hadian, Ruth Brack-Werner, Christine Leib-Mösch

**Affiliations:** Institute of Virology, Helmholtz Zentrum München, German Research Center for Environmental Health, Neuherberg, Germany; Research Unit Cellular Signal Integration, Institute of Molecular Toxicology and Pharmacology, Helmholtz Zentrum München, German Research Center for Environmental Health, Neuherberg, Germany; Department of Hematology and Oncology, University Hospital Mannheim, University of Heidelberg, Mannheim, Germany; Department of Human Genetics, Center of Human and Molecular Biology, Medical Faculty, University of Saarland, Homburg, Germany; Department of Wildlife Diseases, Leibniz Institute for Zoo and Wildlife Research, Berlin, Germany; Assay Development and Screening Platform, Institute of Molecular Toxicology and Pharmacology, Helmholtz Zentrum München, German Research Center for Environmental Health, Neuherberg, Germany

**Keywords:** Human endogenous retroviruses (HERV), Human immunodeficiency virus 1 (HIV-1), HERV transcription profiling, Retrovirus-specific microarray, siRNA

## Abstract

**Background:**

The human genome contains multiple LTR elements including human endogenous retroviruses (HERVs) that together account for approximately 8–9% of the genomic DNA. At least 40 different HERV groups have been assigned to three major HERV classes on the basis of their homologies to exogenous retroviruses. Although most HERVs are silenced by a variety of genetic and epigenetic mechanisms, they may be reactivated by environmental stimuli such as exogenous viruses and thus may contribute to pathogenic conditions. The objective of this study was to perform an in-depth analysis of the influence of HIV-1 infection on HERV activity in different cell types.

**Results:**

A retrovirus-specific microarray that covers major HERV groups from all three classes was used to analyze HERV transcription patterns in three persistently HIV-1 infected cell lines of different cellular origins and in their uninfected counterparts. All three persistently infected cell lines showed increased transcription of multiple class I and II HERV groups. Up-regulated transcription of five HERV taxa (HERV-E, HERV-T, HERV-K (HML-10) and two ERV9 subgroups) was confirmed by quantitative reverse transcriptase PCR analysis and could be reversed by knock-down of HIV-1 expression with HIV-1-specific siRNAs. Cells infected *de novo* by HIV-1 showed stronger transcriptional up-regulation of the HERV-K (HML-2) group than persistently infected cells of the same origin. Analysis of transcripts from individual members of this group revealed up-regulation of predominantly two proviral loci (ERVK-7 and ERVK-15) on chromosomes 1q22 and 7q34 in persistently infected KE37.1 cells, as well as in *de novo* HIV-1 infected LC5 cells, while only one single HML-2 locus (ERV-K6) on chromosome 7p22.1 was activated in persistently infected LC5 cells.

**Conclusions:**

Our results demonstrate that HIV-1 can alter HERV transcription patterns of infected cells and indicate a correlation between activation of HERV elements and the level of HIV-1 production. Moreover, our results suggest that the effects of HIV-1 on HERV activity may be far more extensive and complex than anticipated from initial studies with clinical material.

**Electronic supplementary material:**

The online version of this article (doi:10.1186/s12977-015-0156-6) contains supplementary material, which is available to authorized users.

## Background

Approximately 8–9% of the human genome is composed of endogenous retroviral elements (HERVs). Endogenous retroviruses are found in all phyla and homologues of most HERVs are present among primates, which represents 70 million years of evolutionary time [1]. It is hypothesized that during the course of primate evolution, exogenous progenitors of HERVs inserted themselves into germ-line DNA, where they expanded via reinfection and/or retrotransposition [2,3]. Full-length HERV sequences possess a genomic organization similar to proviruses of exogenous retroviruses. Therefore, HERVs were classified in three major classes according to sequence homologies with the polymerase (*pol*) gene of exogenous animal retroviruses. Class I HERVs show sequence similarities to gammaretroviruses, class II HERVs are related to betaretroviruses and class III HERVs display a limited similarity to spumaviruses [4]. Endogenous homologues to lentiviruses have not been detected in the human genome. There is no evidence to date that infectious HERVs are produced in humans, suggesting their genetic material replicates exclusively as part of their host’s genome. This is in sharp contrast to many other mammals, particularly rodents, in which the lines between endogenous and exogenous retroviruses can become very blurred [5]. Nonetheless, in some instances only a few mutations or recombination events would be required to reconstitute a replication competent provirus in humans [6-8].

Some HERV elements code for individual gene products [9,10] that could be pathogenic. For example, two small regulatory proteins, Rec and Np9, that are encoded by the HERV-K (HML-2) group are suspected to be involved in some human cancers [11-13]. Other examples for potentially pathogenic HERV gene products are the envelope (Env) proteins, which possess fusogenic properties and have been associated with several chronic diseases such as autoimmunity and neurological disorders [14-16].

Most HERVs have been silenced by mutations and/or epigenetic control e.g. methylation of DNA or chromatin modifications. However, they may be reactivated by environmental stimuli such as radiation and chemicals [17-19] or by infectious agents such as exogenous viruses [20-24]. Epstein-Barr virus (EBV) and human herpes virus 6 (HHV-6), for example, have been shown to transactivate expression of a potential HERV-K18 encoded superantigen that stimulates T-cell activation [23,25-29]. This process might be crucial for the establishment of long-term infection by EBV and HHV-6 and play a role in the development of associated diseases. Similarly, expression of HERV-W Env protein has been proposed to be induced by Herpesviridae in patients with multiple sclerosis (MS) and hypothesized to be linked with MS pathogenesis [22]. We have recently shown that human cytomegalovirus (HCMV) activates a number of HERV groups including HERV-K (HML-2) [30]. Because of the association with a multitude of complex diseases, a better understanding of the influence of exogenous viruses on the expression of HERVs in human cells is necessary in various pathogenic contexts.

Lentiviruses and HERVs share many structural and functional features like the LTR as key director and regulator of transcription. The potential complementation of HERV and HIV encoded proteins provides additional interaction points reviewed in [31]. For example, regulatory HIV-1 Rev protein has been shown to substitute for HERV-K (HML-2) Rec (cORF) protein in functional assays [32-34]. On the other hand, HIV-1 infection may compromise intracellular defense mechanisms normally down-regulating HERV activity [35,36]. Data from several reports support an influence of HIV-1 infection on HERV expression. Thus, antibodies against Env proteins of HERV-K102, a member of the HERV-K (HML-2) group, have been found to occur with higher frequency in HIV-1 viraemic patients than in healthy individuals [37]. Another study identified T cells responding to HERV epitopes of various HERV groups in HIV-1 infected individuals [38-41]. Furthermore, HERV-K (HML-2) RNA was reported in peripheral blood mononuclear cells (PBMCs) or plasma of HIV-1 infected patients [42-45].

To gain more insight into the influence of HIV-1 infection on HERV expression on the cellular level, we investigated HERV transcription patterns in persistently HIV-1 infected cell lines with different HIV-1 production levels, in cells infected *de novo* with HIV-1 and in the corresponding uninfected cells. Simultaneous profiling of a large number of HERVs was enabled using a retrovirus-specific DNA chip based on a conserved region within the *pol* gene that covers major HERV groups from all three classes [46,47]. We hypothesized that, if a direct link between HIV-1 and HERV transcription exists, removal of the stimulus (i.e. HIV-1 gene products) should result in a corresponding decrease of the stimulated HERV transcription. Thus, we used siRNAs directed against HIV-1 transcripts as well as a cellular inhibitor of HIV Rev activity to observe their effects on HERV transcription. Furthermore, we identified transcribed HERV-K (HML-2) loci with differential activity in persistently and in *de novo* HIV-1 infected cells. Our data demonstrates up-regulation of several class I and class II HERV groups and links HERV transcription with expression and production of HIV-1 in persistently infected cells.

## Results

### HERV transcription profiles of HIV-1 infected human cell lines with different levels of HIV-1 production

The present study was initiated to explore a possible influence of HIV-1 infection on the transcriptional activities of various HERV groups in HIV-1 infected cells. To this end we used a retrovirus-specific, *pol*-based microarray that allows simultaneous transcription profiling of multiple HERVs and discriminates between 49 HERV subgroups derived from 20 prominent groups representing al three HERV classes [46-48].

**Figure 1:**
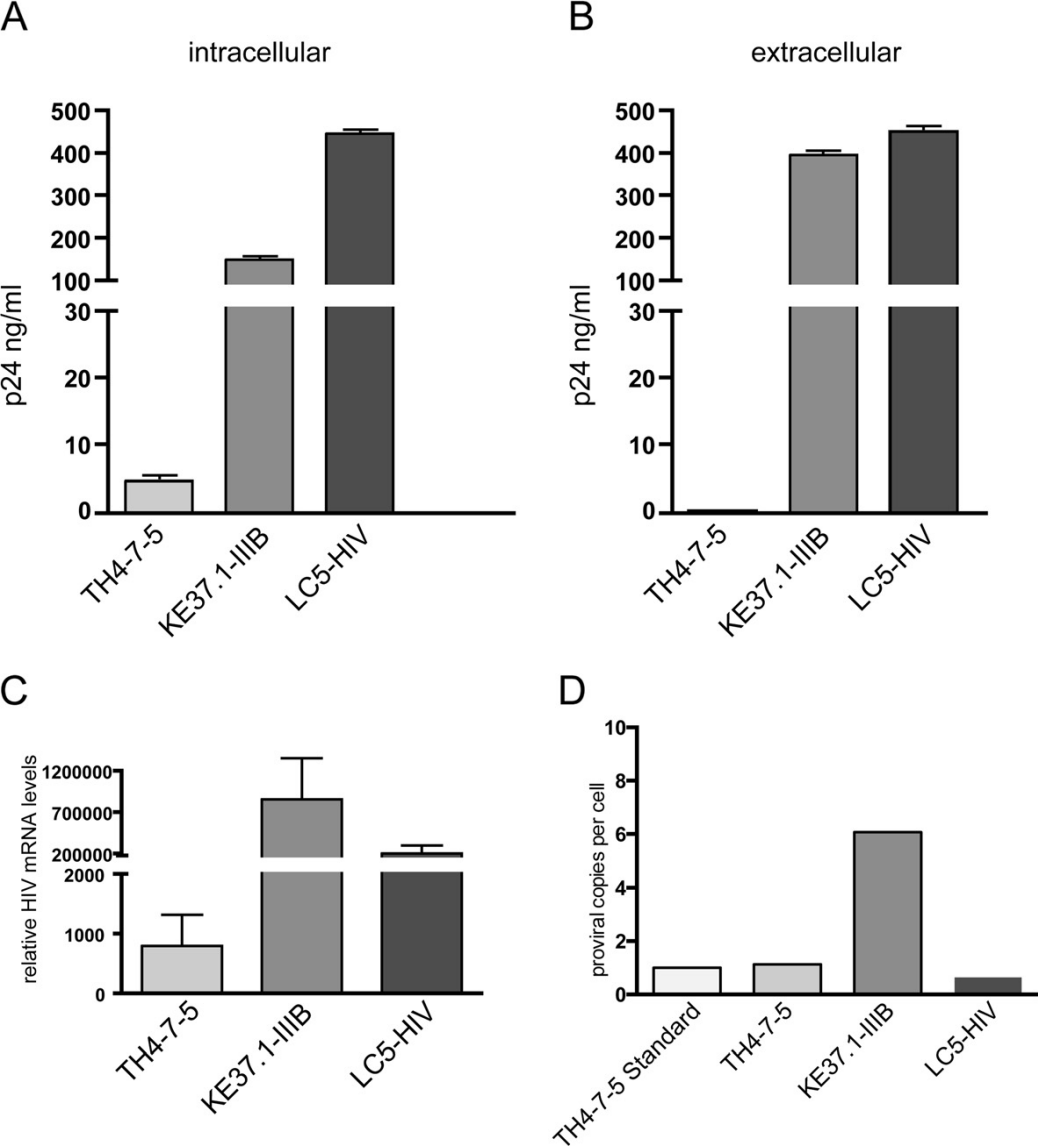
**Productivity of different persistently HIV-1 infected human cell lines. (A)** Intracellular p24-production in the infected cell lines TH4-7-5, KE37.1-IIIB and LC5-HIV. **(B)** Extracellular p24-production in the infected cell lines TH4-7-5, KE37.1-IIIB and LC5-HIV. Quantification of p24 antigen in infected cells and in the supernatant was performed as described [82] using a p24 ELISA. **(C)** HIV-1 transcript levels in the persistently infected cell lines TH4-7-5, KE37.1-IIIB and LC5-HIV. **(D)** Provirus copy numbers of the infected cell lines TH4-7-5, KE37.1-IIIB and LC5-HIV measured with quantitative PCR. Absolute quantification of HIV proviral copies was performed as described in [82].

First we examined three persistently HIV-1 infected cell lines, TH4-7-5, LC5-HIV and KE37.1-IIIB. All persistently infected cell lines contain infectious proviruses. The provirus copy numbers are shown in Figure 1D. We chose these cell lines because they differ in their HIV productivity [49] and thus could be used to determine a possible correlation between levels of HIV-1 production and HERV transcription. The corresponding uninfected cell lines 85HG66 (astrocytes), LC5 (a derivative of HeLa cells [50]) and KE37.1 (T-lymphoma cells), respectively, were used as controls.

The infected cell lines were analyzed for their ability to produce viral proteins by measuring levels of the HIV-1 capsid protein Gag p24 in samples of cell lysates (intracellular p24) and in culture supernatants (extracellular p24) harvested after 24 hours virus production. The three cell lines TH4-7-5, LC5-HIV and KE37.1-IIIB differed clearly in their p24 production levels (Figure 1A). TH4-7-5 cells exhibited the lowest p24 production. The LC5-HIV and KE37.1-IIIB cells produced approximately 10 to 40 fold higher levels of intracellular p24 than the TH4-7-5 cells. In agreement, release of p24 protein was detected only in culture supernatants of LC5-HIV and KE37.1-IIIB cells, but not in supernatants of TH4-7-5 cells (Figure 1B). Using specific PCR primers, we could further show that the infected cell lines produce specific HIV-1 transcripts, whereas the uninfected control cells do not (Figure 1C). This data correlates with the p24 protein levels.

To get a general overview of HERV expression in HIV-1 infected and uninfected cells a retrovirus specific microarray was used. Microarray analysis was conducted in triplicates according to a standardized chip hybridization protocol [46,48,51]. Three house keeping genes served as internal controls for RNA quality. The HIV-1 capture probe served as a control to show that all infected cell lines (KE37.1-IIIB, LC5-HIV, TH4-7-5) are HIV-1 positive and all uninfected cells (KE37.1, LC5, 85HG66) are HIV-1 negative. The microarray consists of 49 representative HERV *pol* (RT) sequences derived from 20 major groups of class I (gammaretrovirus-related), class II (betaretrovirus-related), and class III (spumaretrovirus-related) HERVs [46,47]. Depending on deletions within the targeted *pol* sequence and on sequence variability, the microarray may detect about half up to two third of the elements belonging to a HERV group. The 49 sequences spotted on the chip represent HERV subgroups that are defined by about 20% sequence divergence from each other within the analyzed *pol* region [4,52]. For microarray analysis, conditions were used that require at least 80% sequence identity for hybridization [48,53]. Thus, each HERV subgroup may consist of about 10 to 100 closely related proviral loci with sufficient sequence similarity that individual elements cannot be distinguished. Depending on the size of a subgroup and its transcriptional activity, one or more transcribed loci may hybridize to one spot of the microarray, and in a few cases cross-hybridization between related subgroups is observed. False positive signals cannot be ruled out completely, but were minimized by amplifying the hybridization probe with HERV-specific primers before microarray hybridization. Despite of the limitations this method allows a fast and comprehensive screening of overall HERV activity in a cell type.

**Figure 2:**
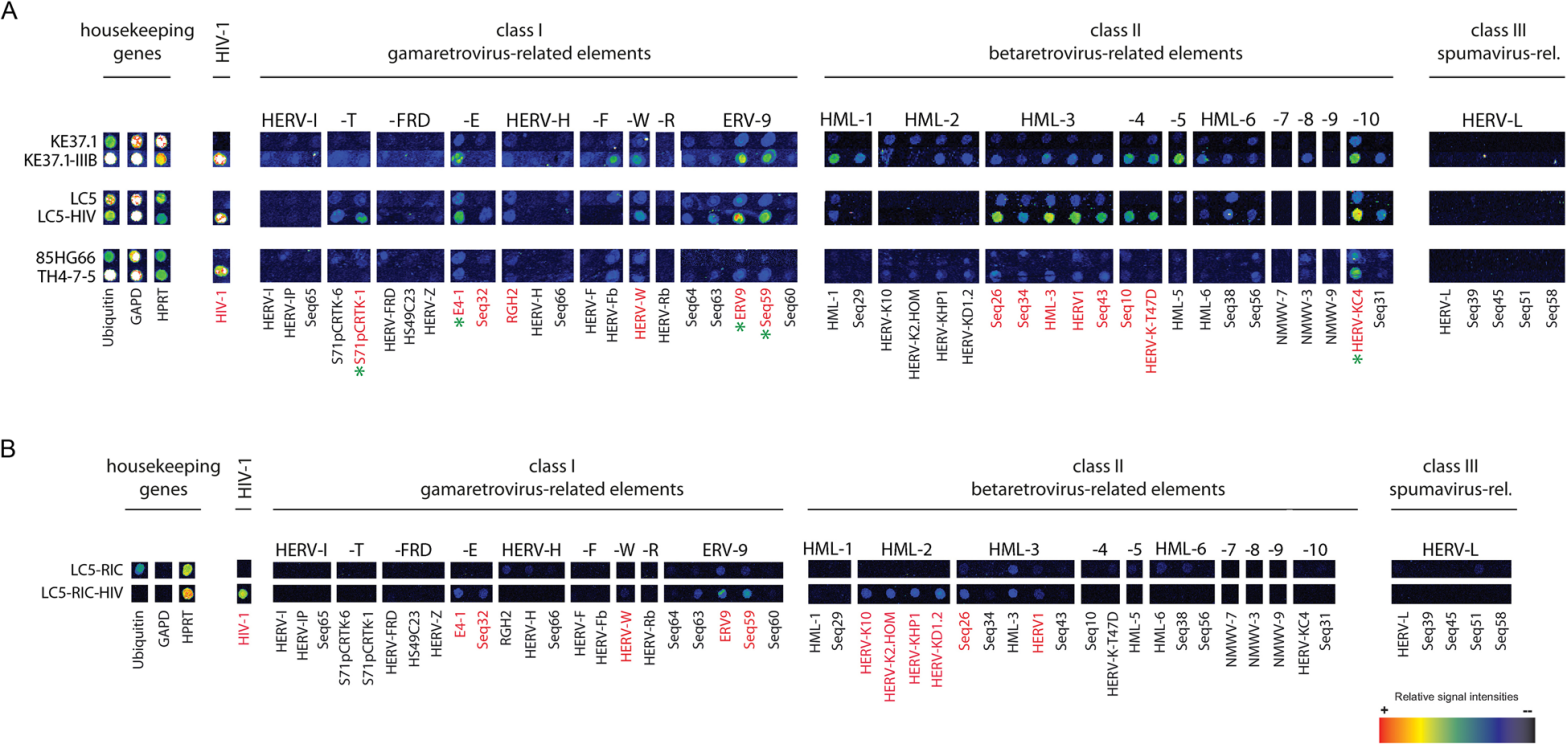
**HERV transcription profiles of HIV-1 infected cell lines examined by a retrovirus-specific microarray.** False color mapping was used for image visualization. The house keeping genes ubiquitin, glycerinaldehyde-3-phosphate-dehydrogenase (GAPDH) and hypoxanthine-guanine phosphoribosyltransferase (HPRT) served as a quality control and internal standard. HIV-1 oligonucleotides are also spotted on the chip as a positive control and to demonstrate HIV-1 infection. HERVs are grouped in class I, II and III elements. It should be noted that each positive spot on the microarray can represent multiple HERV proviruses of one multicopy HERV subgroup with sufficient sequence similarity that individual elements cannot be distinguished. **(A)** Comparison of persistently HIV-1 infected cell lines with the corresponding uninfected cell lines. Up-regulated HERV subgroups are indicated by red letters. HERV subgroups marked with a green asterisk were additionally analyzed by qRT-PCR. **(B)** Comparison of *de novo* HIV-1 infected LC5-RIC cells with uninfected cells.

Figure 2A displays an alignment of the hybridization patterns obtained with RNA from persistently HIV-1 infected cell lines compared to the corresponding uninfected cells. Members of 7 HERV groups, HERV-T, HERV-E, HERV-W, ERV-9, HML-3, HML-4 and HML-10, belonging to class I and II HERVs were found up-regulated in virus producing cells when compared to uninfected control cells (marked in red in Figure 2A). The more ancient class III HERV elements remained unaffected in all cell lines. A significant increase of HERV-K (HML-2) transcripts, previously associated with HIV-1 infection in patients [38,42] was not detected in any of the persistently infected cell lines by microarray analysis. Up-regulation of HERV-F, HML-1 and HML-5 was observed only in the HIV-1 infected lymphocytic cell line KE37.1-IIIB. Comparison of the HERV transcription profiles of the three infected cell lines with each other suggests that the up-regulation of HERV transcription may be connected to HIV-1 production levels.

To analyze the influence of *de novo* HIV-1 infection and to determine, whether HERV transcription patterns in *de novo* and persistently infected cells differ, we infected LC5 cells expressing a fluorescent HIV reporter (LC5-RIC) with the HIV-1 patient isolate P-891 described in [54,55]. The *de novo* HIV-1 infected cells showed a HERV transcription pattern partially similar to that of persistently infected LC5 cells. Both, *de novo* and persistently infected cells displayed increased transcription of HERV-E and ERV9 subgroups (Figure 2A and B). However, enhanced transcription of members of groups HERV-T, HERV-W, HML-4 and HML-10 was observed only in persistently HIV-1 infected LC5 cells but not in *de novo* infected cells. Stimulation of HERV-K (HML-3) transcription was much weaker in *de novo* infected cells than in persistently infected cells. On the other hand, the *de novo* HIV-1 infected LC5-RIC cells showed a stronger activation of HERV-K (HML-2) transcription than persistently infected cells.

### Quantitative reverse transcriptase PCR confirms differential transcription of HERVs in HIV-1 infected and uninfected cells

**Figure 3:**
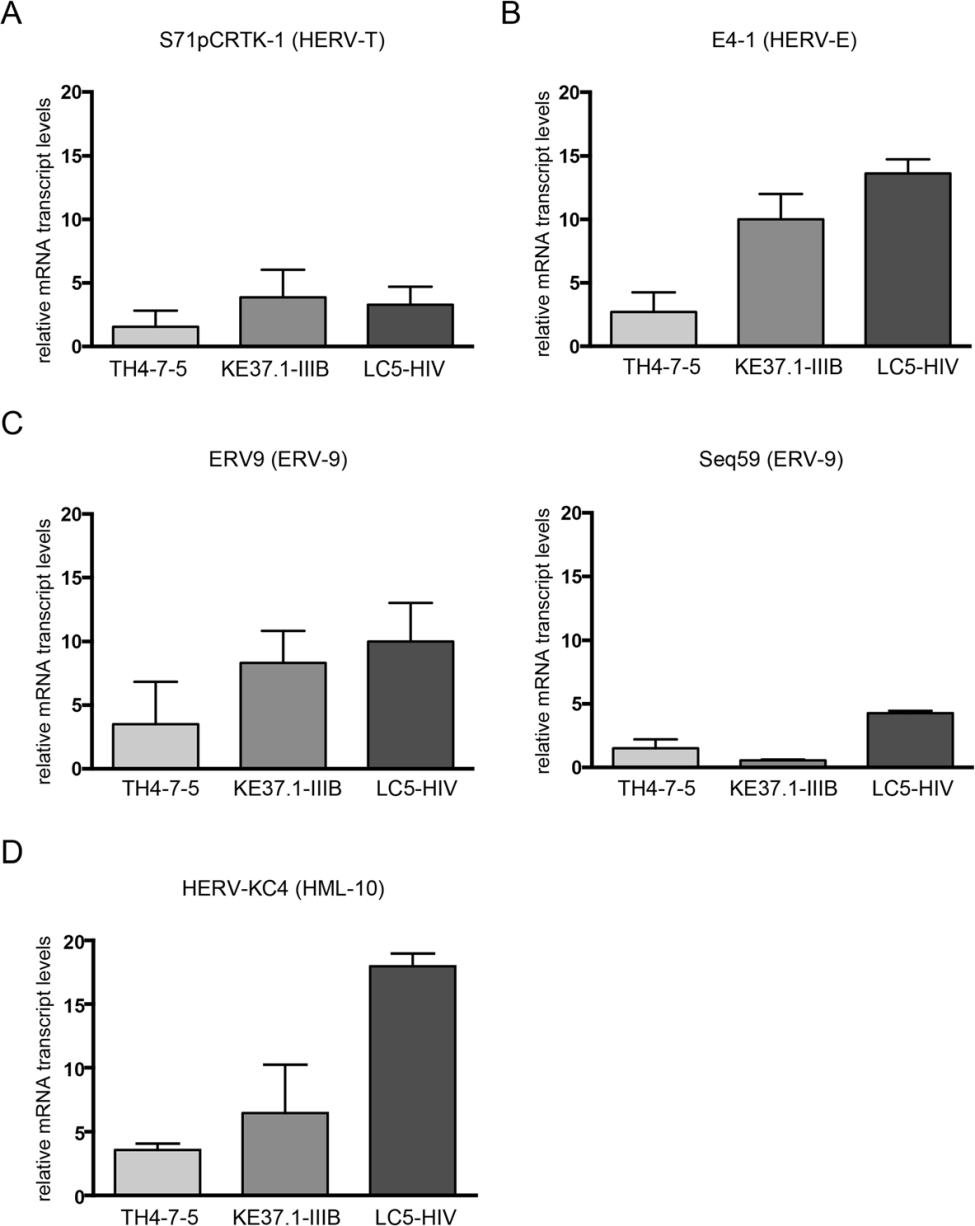
**Relative transcriptional activity of selected HERV subgroups.** Relative transcript levels of **(A)** HERV taxa S71pCRTK-1 (group HERV-T), **(B)** E4-1 (group HERV-E), **(C)** ERV9 and Seq59 (both group ERV9) and **(D)** HERV-KC4 (group HERV-K (HML-10)) were determined by qRT-PCR. The Y-axis shows the x-fold relative expression of HERV-transcripts in infected cells referred to uninfected cells. Relative transcription was quantified according to [85] and normalized to RNA Polymerase II (RPII) transcript levels. Standard errors for triplicate experiments are indicated.

Differences in the transcription levels of five representative HERV subgroups in infected and non-infected cells were subsequently analyzed by qRT-PCR using primers that bind specifically to the *pol* region of the up-regulated HERVs. These primers are located in a region of the reverse transcriptase gene that exhibits only marginal similarity among HERV-taxa with one primer matching the sequence of the corresponding microarray capture probes [46,48]. Figure 3 shows the relative transcript levels of the selected HERV taxa S71pCRTK-1 (group HERV-T), E4-1 (group HERV-E), ERV9 and Seq59 (both group ERV9), and HERV-KC4 (group HML-10) in the HIV-1 infected cells compared to the uninfected control cells. Transcript levels of HERV subgroups S71pCRTK-1 (Figure 3A) and Seq59 (Figure 3C) were only slightly increased, whereas levels of E4-1 (Figure 3B), ERV9 (Figure 3C) and HERV-KC4 (Figure 3D) were up to 15fold higher in HIV-1 infected cells than in uninfected cells. This result was in agreement with the microarray data (Figure 2A). The cell lines with high HIV-1 production (KE37.1-IIIB and LC5-HIV) demonstrated higher HERV transcription than the non-productive TH4-7-5 cells. Thus, the results obtained by two independent methods indicate that persistent HIV-1 infection increases transcription of at least one member of each of 5 HERV subgroups and that transcription levels of these HERVs are related to HIV-1 production.

### HIV-1-specific siRNAs reverse HERV up-regulation

To demonstrate that the observed alterations are indeed associated with HIV-1 infection, we investigated if the up-regulated HERV transcription could be reversed by silencing of HIV-1 transcription using RNA interference assays. The siRNAs against HIV-1 transcripts were selected according to ter Brake and Berkhout [56]. An overview of the HIV-1 transcripts, which are silenced by the selected siRNAs is shown in Additional file 1A. The siRNA sigag is predicted to silence all *gag* and *pol* transcripts, sitat/rev all transcripts except *nef,* and sienv together with sinef all HIV-1 transcripts. HIV-1 contains three major RNA splice variants coding for all proteins. These RNAs harbor overlapping sequences making it impossible to design siRNAs that are unique for knockdown of individual proteins. An exception is sigag targeting a *gag* sequence that is exclusively present in unspliced, late mRNA. Contrary to expectation however, sigag has been shown to down-regulate all transcripts, possibly due to nuclear targeting of HIV-1 mRNAs prior to splicing [56]. As control, a non-silencing siRNA was used, and an experiment without siRNA (mock experiment) was performed to control for unspecific silencing effects by siRNAs in general and by the transfection reagent, respectively. The capacity of the siRNAs to inhibit HIV-1 production was tested in LC5-HIV cells. All HIV-1-specific siRNAs efficiently decreased levels of full-length HIV-1 transcripts (Additional file 1B) and of the Gag p24 protein (data not shown) 72 hours after siRNA transfection. The knock-down effect of these siRNAs on spliced HIV-1 transcript was not tested in this study, but has been demonstrated previously [56].

**Figure 4:**
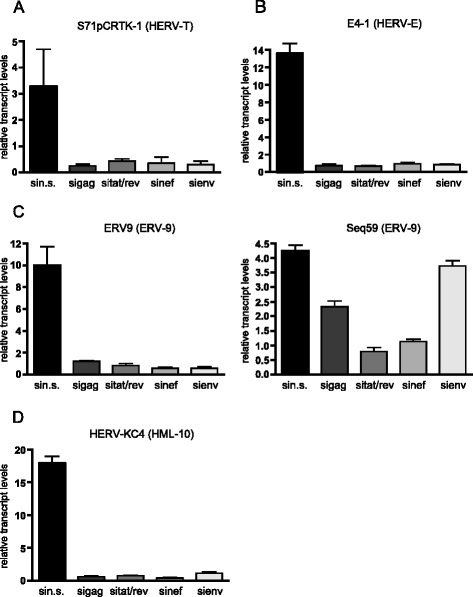
**Down-regulation of HIV-1 induced HERV activity by siRNAs targeting HIV-1 transcripts.** LC5-HIV cells were treated with the RNAiFect transfection reagent (mock), non-silencing siRNAs (sin.s.) or with siRNAs against *gag* (sigag), *rev* (sirev), *nef* (sinef) and *env* (sienv). Relative transcript levels of **(A)** HERV taxa S71pCRTK-1 (group HERV-T), **(B)** E4-1 (group HERV-E), **(C)** ERV9 and Seq59 (both group ERV9) and **(D)** HERV-KC4 (group HERV-K (HML-10)) were determined by qRT-PCR. The Y-axis shows the x-fold relative HERV transcript levels in LC5-HIV cells transfected with non-silencing siRNA (sin.s) and HIV-1-specific siRNAs (sigag, sitat/rev, sinef, sienv) referred to uninfected control cells. The data were normalized to the house-keeping gene RNA Polymerase II (RPII). Standard errors for triplicate experiments are indicated.

The five HERV taxa S71pCRTK-1, E4-1, ERV-9, Seq59 and HERV-KC4 activated by HIV-1 in previous experiments were analyzed by qRT-PCR 72 hours after transfection of the siRNAs. Figure 4 shows the relative transcription levels of the HERV subgroups in the HIV-1 infected HeLa cells (LC5-HIV) transfected with specific siRNAs against *gag, rev, nef* and *env* compared to control cells transfected with non-silencing siRNA. Knockdown of HIV-1 in LC5-HIV cells resulted in a loss of HERV transcription in four of five HERV subgroups (Figure 4A-D). Only Seq59, a subgroup of ERV9 elements (Figure 4C), appears to be only partially diminished. Thus, we can show that knockdown of HIV-1 transcripts by RNAi reverses activation of specific HERV elements in infected cells. Moreover, overexpression of a natural inhibitor of HIV-1 production, the Rev-interacting human protein family (Risp) [57], was found to reverse HERV activation in HIV-1 infected LC5 cells (Additional file 2). Taken together, our results indicate that HIV-1 infection is associated with the activation of several taxa of class I and class II HERVs.

### Differential HERV-K (HML-2) transcription in *de novo* and persistently HIV-1 infected cells

Plasma of HIV-1 infected individuals has been reported to contain elevated levels of HERV-K (HML-2) RNA [38,42,43]. In our microarray experiments we observed a significant increase of HERVK (HML-2) transcription only in *de novo* infected cells but not in the persistently infected cell lines (Figure 2) suggesting that an up-regulation of group HERV-K (HML-2) proviruses occurs preferentially within a short period after infection. To investigate, which HERV-K (HML-2) loci contribute to the observed up-regulation, we cloned and sequenced HERV-K (HML-2) *gag* transcripts and combined the data with qRT-PCR analysis.

**Figure 5:**
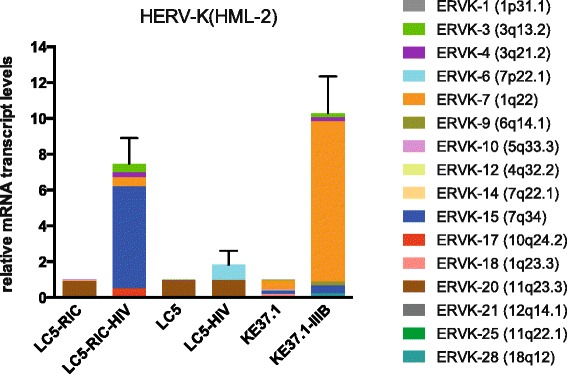
**Transcriptional activity of HERV-K (HML-2) proviral loci in** ***de novo*** **and persistently infected cells.** The Y-axis shows the x-fold relative expression of HERV-K (HML-2) *gag* transcripts in infected cells referred to uninfected cells. Relative transcription was quantified according to [85] and normalized to RPII transcript levels. HERV-K (HML-2) transcripts were amplified using *gag-*specific primers, cloned, sequenced and assigned to proviral loci as described previously [58]. For each HERV-K (HML-2) locus the relative cloning frequency of cDNA is shown as percentage of the total HERV-K (HML-2) transcription level determined by qRT-PCR.

Since most commonly used primers designed to amplify HERV-K (HML-2) co-amplify HML-3 sequences [42], we generated new *gag* derived primers for qRT-PCR that are highly specific and amplify exclusively members of the HERV-K (HML-2) group. Analysis by qRT-PCR revealed an approximately 8fold higher overall level of HERV-K (HML-2) transcription in *de novo* HIV-1 infected LC5-RIC cells compared to uninfected cells, whereas in persistently infected LC5 cells the HERV-K (HML-2) transcript level increased only about 1.8fold above the transcript level in uninfected cells [58] (Figure 5). This data suggests that up-regulation of HERV-K (HML-2) proviruses may be largely restricted to *de novo* HIV-1 infection.

To investigate the contribution of individual proviral loci to increased HERV-K (HML-2) transcription in HIV-1 infected LC5-RIC cells, HERV-K (HML-2) transcripts were amplified using the same HERV-K (HML-2)-specific forward primer as applied for qRT-PCR in combination with a downstream located reverse primer amplifying an about 650 bp *gag* gene derived PCR product. This *gag* region contains a sufficient amount of nucleotide differences to allow a clear discrimination between different proviral loci [58].

Sequence analysis of cloned RT-PCR products derived from uninfected cells showed predominant transcription of a single HERV-K (HML-2) locus on chromosome 11q23.3, ERVK-20. Transcripts from this locus comprise 94% and 85% of total transcripts in LC5 and LC5-RIC cells, respectively (Table 1, Figure 5). In addition, different HERV-K (HML-2) loci show minor activity in both cell lines. LC5-RIC cells are a subline of LC5 cells generated by introducing the CD4 receptor gene and a DsRED1 reporter, and therefore may show alterations of HERV transcription compared to the original LC5 cells. In persistently infected LC5-HIV cells an additional HERV-K (HML-2) provirus on chromosome 7p22.1, ERVK-6, is activated that presumably accounts for the approximately 1.8fold increase of HERV-K (HML-2) transcription observed by qRT-PCR (Figure 5). Interestingly, ERVK-6 has intact open reading frames for all retroviral genes [59]. In contrast to the single locus up-regulated in persistently infected cells, analysis of *de novo* HIV-1 infected LC5-RIC cells revealed five transcriptionally active HML-2 loci with ERV-K15 on chromosome 7q34 comprising about 75% of total transcripts (Table 1, Figure 5). In the T-lymphoma cell line KE37.1 the basal HML-2 transcription pattern (Table 2, Figure 5) resembles more that obtained with PBMCs from healthy persons (Additional file 3) than that of the epithelial LC5 cell line (Table 1, Figure 5). Persistently infected KE37.1-IIIB cells show predominant activation of a locus on chromosome 1q22, ERK-7 (Table 2, Figure 5). Taken together, these data demonstrate that activation of HERV loci by HIV-1 is also cell type-specific and may depend on epigenetic control of HERV loci differing in various cell types.

**Table 1:** **Differential HERV-K (HML-2) transcription in** ***de novo*** **and persistently infected LC5 cells**

**Provirus** ^**a**^	**Chromosome band**	**Location of amplicon in genome** ^**b**^	**Number of clones (% cloning frequency)**			
			**LC5-RIC**	**LC5-RIC-HIV** ^**c**^	**LC5**	**LC5-HIV** ^**d**^
ERVK-7^e^ (c1_B, K102, K50a)	1q22	153870020–153870613	-	2 (7.1)	-	-
ERVK-18 (c1_C, K110, K18)	1q23.3	158928993–158929564	1 (2.1)	-	-	-
ERVK-3^e,f^ (c3_B, K106, K68)	3q13.2	114232740–114233333	-	2 (7.1)	2 (2.2)	-
ERVK-4^e^ (c3_C, K (I))	3q21.2	127093474–127094067	1 (2.1)	1 (3.6)	2 (2.2)	-
ERVK-6^e,f^ (c7_A, K (HML-2. HOM), K108)	7p22.1	4604296–4604932	-	-	-	44 (47.3)
		4595814–4596407^g^				
ERVK-14^e^ (c7_B)	7q22.1	104179245–104179826	1 (2.1)	-	-	-
ERVK-15 (c7_C, K (OLDAC004979))	7q34	141101009–141101702	-	21 (75.0)	1 (1.1)	-
ERVK-17^e^ (c10_B)	10q24.2	101576433–101577026	-	2 (7.1)	-	-
ERVK-25^e,f^ (c11_A, K36, K118)	11q22.1	101072651–101073244	3 (6.4)	-	-	-
ERVK-20 (c11_B, K37)	11q23.3	118103858–118104446	40 (85.1)	-	84 (94.4)	49 (52.7)
ERVK-21 (K (C12), K41, K119)	12q14.1	58728458–58729051	1 (2.1)	-	-	-
Total number of clones			47 (100)	28 (100)	89 (100)	93 (100)

^a^Designation according to [87], aliases are in parentheses.

^b^According to the March 2006 assembly (NCBI36/hg18) of the human genome sequence at the Human Genome Browser [88].

^c^ *de novo* infected LC5-RIC cells.

^d^persistently infected LC5 cells.

^e^Human-specific HERV-K (HML-2) proviruses [89].

^f^Polymorphic in the human genome [89].

^g^The human reference genome sequence harbors a tandem provirus [90]. Due to the lack of sufficient sequence differences the detected cDNA sequence may stem from one or the other provirus.

**Table 2:** **Differential HERV-K (HML-2) transcription in the uninfected and persistently infected human T-lymphoma cell line KE37.1**

**Provirus** ^**a**^	**Chromosome band**	**Location of amplicon in genome** ^**b**^	**Number of clones (% cloning frequency)**	
			**KE37.1**	**KE37.1-IIIB** ^**c**^
ERVK-1^d,e^ (c1_A, K116, K4)	1p31.1	75617005–75617599	1 (2.9)	-
ERVK-7^d^ (c1_B, K102, K50a)	1q22	153870019–153870613	18 (51.4)	40 (87.0)
ERVK-18 (c1_C, K110, K18)	1q23.3	158928865–158929429	7 (20.0)	-
ERVK-3^d,e^ (c3_B, K106, K68)	3q13.2	114232739–114233333	1 (2.9)	1 (2.2)
ERVK-4^d^ (c3_C, K (I))	3q21.2	127093473–127094067	-	-
ERVK-12 (c4_A, K5)	4q32.2	161800867–161801461	1 (2.9)	-
ERVK-10^d^ (K107, K10, c5)	5q33.3	156024233–156024827	1 (2.9)	1 (2.2)
ERVK-9^e^ (c6_A, K109, envK4)	6q14.1	78490569–78491163	-	1 (2.2)
ERVK-15 (c7_C, K (OLDAC004979))	7q34	141101012–141101702	6 (17.1)	2 (4.3)
ERVK-28	19q12	32827928–32828522	-	1 (2.2)
Total number of clones			35 (100%)	46 (100%)

^a^Designation according to [87], aliases are in parentheses.

^b^According to the March 2006 assembly (NCBI36/hg18) of the human genome sequence at the Human Genome Browser [88].

^c^human T-lymphoma cell line KE37.1 persistently infected with HIV-1IIIB/LAI.

^d^Human-specific HERV-K (HML-2) proviruses [89].

^e^Polymorphic in the human genome [89].

**Figure 6:**
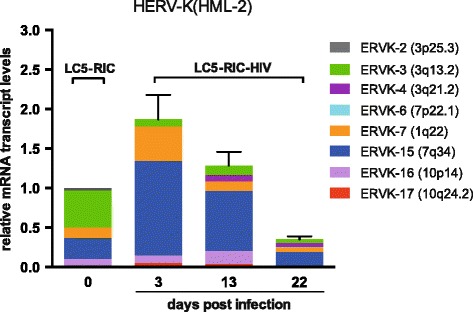
**Time course of HERV-K (HML-2) in** ***de novo*** **HIV-1 infected LC5-RIC cells.** The Y-axis shows the x-fold relative expression of HERV-K (HML-2) *gag* transcripts in infected cells referred to uninfected cells. RNA was isolated from LC5-RIC cells prior to infection and from cells infected with HIV-1IIIB/LAI at day 3, 13 and 22. Relative transcription was quantified according to [85] and normalized to RPII transcript levels. HERV-K (HML-2) transcripts were amplified using *gag*-specific primers, cloned, sequenced and assigned to proviral loci as described previously [58]. For each HERV-K (HML-2) locus the relative cloning frequency of cDNA is shown as percentage of the total HERV-K (HML-2) transcription level determined by qRT-PCR.

To study the transcriptional activity of HERV-K (HML-2) proviruses in the time course of *de novo* HIV-1 infection, a subclone of the original LC5-RIC cells was infected with HIV-1IIIB/LAI. This subclone yields a high proportion of infected cells (Additional file 4) superseding the removal of uninfected cells by cell sorting. Although the LC5-RIC subclone shows a basal HERV-K (HML-2) transcription pattern that differs from that of the original LC5 as well as the parental LC5-RIC cells, infection with HIV-1 leads to a very similar HERV-K (HML-2) activation pattern (Table 3, Figure 6) compared to that of the infected parental LC5-RIC cells (Table 1, Figure 5). As in these cells, locus ERVK-15 (7q34) shows the highest increase of transcriptional activity. Furthermore, minor activation was also detected for ERVK-7 (1q22), which has been shown to be preferentially transcribed in persistently infected KE37.1-IIIB cells. After thirteen days a decline of HERV-K (HML-2) activity and a decrease of HIV-1 provirus copies was observed (Figure 6, Additional file 4), presumably due to HIV-1 cytotoxicity.

**Table 3:** **Time course of HERV-K (HML-2) transcription in HIV-1 infected LC5-RIC cells**

**Provirus** ^**a**^	**Chromosome band**	**Location of amplicon in genome** ^**b**^	**Number of clones (% cloning frequency)**			
			**Day-0**	**Day-3**	**Day-13**	**Day-22**
ERVK-7^c^ (c1_B, K102, K50a)	1q22	153870020–153870613	4 (13.3)	9 (23.1)	3 (9.4)	6 (16.7)
ERVK-2 (c3_A, K11)	3p25.3	9869046–9869639	1 (3.3)	-	-	-
ERVK-3^c,d^ (c3_B, K106, K68)	3q13.2	114232740–114233333	14 (46.7)	2 (5.1)	3 (9.4)	5 (13.8)
ERVK-4^c^ (c3_C, K (I))	3q21.2	127093474–127094067	-	-	2 (6.3)	5 (13.8)
ERVK-6^c,d^ (c7_A, K (HML-2. HOM), K108)	7p22.1	4604296–4604932	-	-	-	1 (2.8)
		4595814–4596407^e^				
ERVK-15 (c7_C, K (OLDAC004979))	7q34	141101009–141101702	8 (26.7)	25 (64.1)	19 (59.4)	19 (52.8)
ERVK-16 (c10_A, M3.8)	10p14	6913372–6913968	3 (10.0)	2 (5.1)	4 (12.5)	-
ERVK-17^c^ (c10_B)	10q24.2	101576433–101577026	-	1 (2.6)	1 (3.1)	-
Total number o f clones			30 (100)	39 (100)	32 (100)	36 (100)

^a^Designation according to [87], aliases are in parentheses.

^b^According to the March 2006 assembly (NCBI36/hg18) of the human genome sequence at the Human Genome Browser [88].

^c^Human-specific HERV-K (HML-2) proviruses [89].

^d^Polymorphic in the human genome [89].

^e^The human reference genome sequence harbors a tandem provirus [90]. Due to the lack of sufficient sequence differences the detected cDNA sequence may stem from one or the other provirus.

In contrast to HERV-K (HML-2), the transcription pattern of HERV-K (HML-3) elements detected with HML-3 *env*-specific primers is similar in *de novo,* persistently infected and in uninfected LC5 cells (Additional file 5). Transcripts of the two prevailing HERV-K (HML-3) loci 1q21.2 and 3q21.3 were found with cloning frequencies in the range of 40–50% and 17–30%, respectively, in persistently and *de novo* infected cells, as well as in uninfected cells. The 10 additional, minor active HERV-K (HML-3) loci likewise showed only slight transcription variation suggesting a less differentiated influence of HIV-1 infection on the HERV-K (HML-3) group.

### Cellular transcription factors are potential mediators of HERV activation

To address the question, whether HERV transcription may be up-regulated via activation of cellular transcription factors by HIV-1 gene products, we stimulated Jurkat T-cells by treatment with PMA/Ionomycin or by CD3/CD28 antibody ligation [60]. PMA possesses structural similarity to diacylglycerol (DAG) and can therefore activate PKCθ and thus NF-κB in T cells. Ionomycin induces Ca2^+^ influx from intracellular Ca2^+^ storage compartments and therefore mainly influences the activation of NFAT. Crosslinking of the co-stimulatory receptor CD28 and the CD3 subunits of the T cell receptor by specific primary and secondary antibodies mimics the receptor aggregation, which under physiological conditions is induced by the T cell/APC (antigen presenting cell) contact. This leads to activation of antigen-receptor-specific signaling cascades.

**Figure 7:**
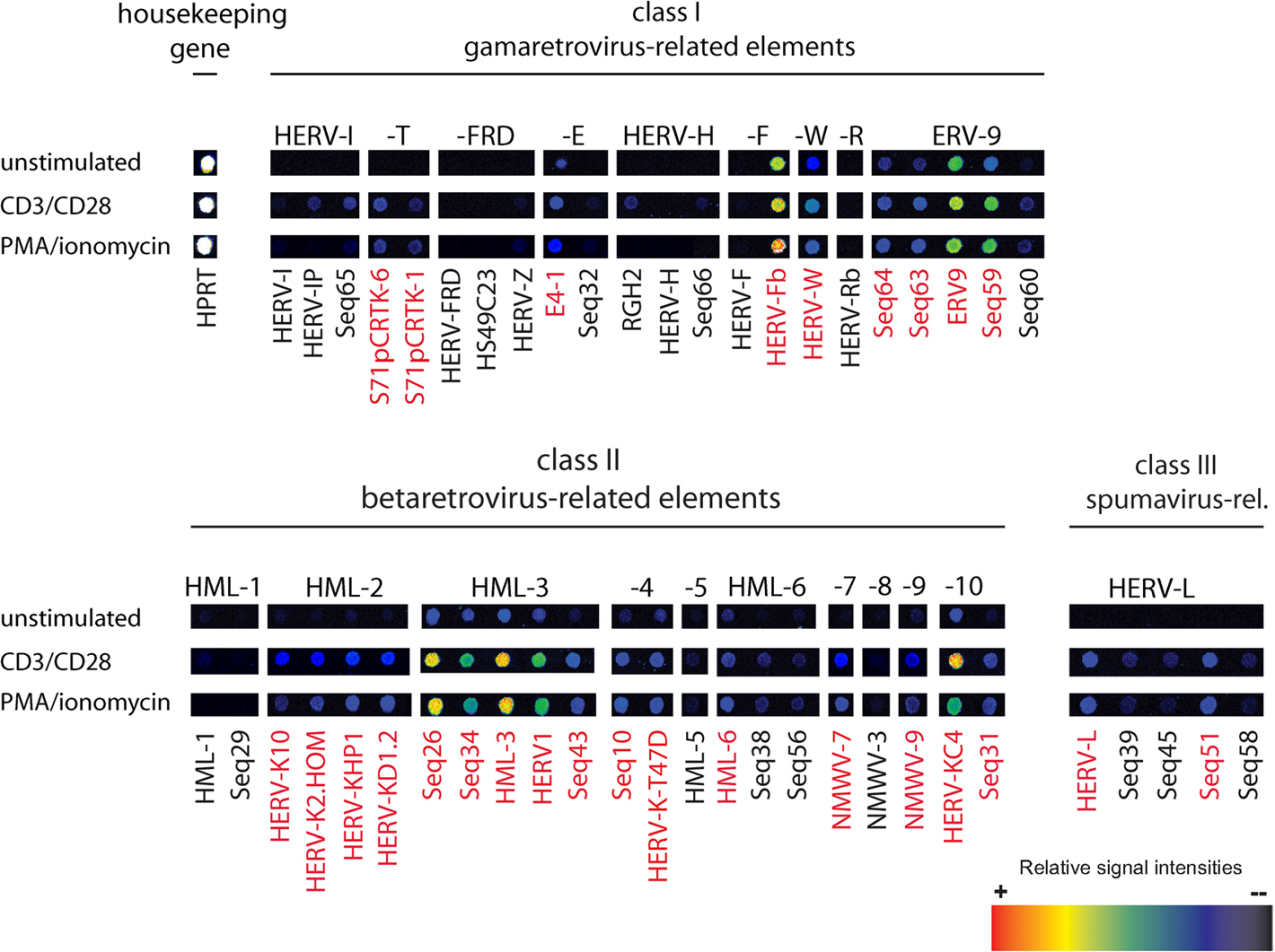
**HERV transcription profiles of PMA/ionomycin and CD3/CD28 stimulated Jurkat T-cells.** False color mapping was used for image visualization. The house keeping gene HPRT served as a quality control and internal standard. Up-regulated HERV subgroups are indicated by red letters.

Using RetroArray analysis, the HERV activation pattern of stimulated T-cells was compared with that obtained after HIV-1 infection (Figure 7 and 2). A variety of HERV groups derived from all three HERV classes was found to be up-regulated after addition of both stimuli, PMA/Ionomycin and CD3/CD28 (Figure 7). Interestingly, the transcription pattern in stimulated T-cells includes HERV groups also affected in *de novo* (HERV-E, ERV9, HERV-K (HML-2), HERV-K (HML-3)) as well as in persistently HIV-1 infected cells (HERV-T, ERV9, HERV-K (HML-3), HERV-K (HML-4), HERV-K (HML-10)). Since IL2 is a major target of the transcription factors induced by PMA/Ionomycin or CD3/CD28, we verified successful stimulation of the cells by measuring IL2 mRNA (Additional file 6). The data suggests that major transcription factors like NF-κB, NFAT and AP1 may be involved in the activation of at least some HERV taxa in HIV-1 infected cells.

## Discussion

Interactions between HIV and endogenous retroviruses in infected patients may have implications for intracellular defense mechanisms and immune response and thus may influence the pathogenic process in many ways (reviewed in [31]). However, the study of HIV-HERV interactions in clinical samples from HIV-1 infected individuals is associated with several problems. A major difficulty is the limited amount of HIV-1 infected cells. Analysis of the mean HIV-1 integrated DNA in CD4^+^ T-cells from individuals under antiretroviral therapy (ART) revealed less than 1100 copies per 10^6^ cells [61,62], indicating infection frequencies < 0.2% of CD4^+^ T-cells. An additional problem is that basal HERV transcription in PBMCs may mask activation of HERVs by HIV-1. Several class I and class II HERV groups are typically transcribed in blood cells of healthy people [46,47,63]. In particular, transcripts of the HERV-K (HML-2) group have been identified in PBMCs derived from healthy individuals in several studies [64-66] in agreement with our recent results revealing transcription of at least 12 different HERV-K (HML-2) loci in PBMCs from healthy donors (Additional file 3). Finally, HERV transcription profiles may exhibit among individual variability independent of HIV-1 infection. In every tissue type investigated so far, we detected HERVs differentially transcribed between individuals in addition to the constitutively active HERV groups [51,53]. Therefore, blood samples of patients before infection with HIV-1 would be needed for an exact and reliable assessment of HIV-1 mediated HERV activation and these are usually not available.

To overcome these difficulties, we conceived a systematic study using well-defined cell culture systems consisting of various HIV-1 infected cell lines. This approach offers a much more homogenous genetic background for identification of HIV-1 associated changes in HERV transcription pattern than clinical samples and provides the appropriate controls to evaluate expression of HERVs in the absence of HIV-1 infection. We compared HERV transcription levels in three different persistently infected cell lines with the respective uninfected cells. In addition, we investigated the influence of HIV-1 on HERV activity in *de novo* infected cells. Activated transcription of several class I and class II HERV groups was demonstrated by RetroArray analysis and confirmed by qRT-PCR for five selected HERV subgroups (S71pCRTK-1, HERV-E (4–1), ERV-9, ERV9 Seq59 and HERV-KC4). The extent of HERV up-regulation roughly correlated with production levels of HIV-1 structural proteins in each cell line. Importantly, activation of HERV transcription was reversed by knock-down of HIV-1 transcript levels with HIV-1-specific siRNAs. These results indicate that HIV-1 infection can activate transcription of at least five HERV subgroups.

RetroArray analysis also showed considerable activation of HERV-K (HML-2) elements in *de novo* infected LC5 cells compared to persistently infected cell lines. This indicates that immediate responses of HERVs to HIV-1 infection may differ from long-term effects. Since the youngest and most active HERVs belong to the HERV-K (HML-2) group and HERV-K (HML-2) proviruses were reported previously to be associated with HIV-1 infection (reviewed in [31]), we examined HERV-K (HML-2) in more detail. A preferential activation of two loci (ERVK-7 and -15) on chromosomes 1q22 and 7q34 was observed in *de novo* infected LC5 cells, as well as in persistently infected KE37.1 cells, resulting in an 8fold to 10fold increase of overall HERV-K (HML-2) transcription. During three weeks after *de novo* infection of LC5 cells only slight alterations of the HERV-K (HML-2) transcription pattern were observed Persistently infected LC5 cells, however, showed a completely different HERV-K (HML-2) transcription pattern. The HERV loci activated in *de novo* infected cells are turned off, but a new provirus, ERVK-6 (7p22.1), is transcribed in addition to the ERVK-20 locus active in uninfected cells. The distinct difference between HERV-K (HML-2) loci transcribed in *de novo* and in persistently infected cells suggests different modes of interaction with HIV-1.

In contrast to the multiple HERV-K (HML-2) loci previously reported to be activated in HIV-1 infected patients, only few HML-2 loci are influenced by HIV-1 in persistently and *de novo* infected LC5 cells, as well as in the T-lymphoma cell line KE37.1. Contreras-Galindo *et al.* report detection of *env* transcripts derived from 34 different HERV-K (HML-2) loci in plasma of 7 HIV-1 patients [44]. This inconsistency might be explained by the use of LC5 cells in our *in vitro* experiments, where HML-2 transcription seems to be generally more restricted than in PBMCs (Additional file 3, see also ref. [66]), but also in KE37.1 cells, which display a basal HERV-K (HML-2) transcription pattern very similar to that of PBMCs of healthy persons, only 6 active loci were detected in persistently infected cells. However, also technical problems such as occurrence of genomic DNA from lysed cells in plasma of patients may influence the examination of blood samples [67,37]. A modest increase of class II (HERV-K) elements was also detected by Lefebvre *et al.* in SupT1 cells infected with a VSV-G pseudotyped HIV vector using SAGE sequencing of the whole transcriptome, but the obtained sequences could not be assigned to specific HERV-K loci or groups such as HERV-K (HML-2) in this study [68].

There are several possible explanations how HIV-1 infection may enhance HERV transcription in infected cells. First it may be a side effect that occurs when HIV-1 impairs cellular antiretroviral functions [69,70]. Viruses use various strategies to communicate with host cells and antagonize cellular restrictions. For example, HIV-1 Vif neutralizes the cytidine deaminase activity of APOBEC3G, and Vpu and Nef may counteract Tetherin. There is evidence that APOBEC proteins have contributed to hypermutation of HERV-K (HML-2) elements before they were genetically fixed in the human genome [71,72]. However, restriction by APOBEC3G as well as Tetherin would require *de novo* replication of HERVs and reintegration of mutated HERV sequences, which is highly unlikely.

Contreras-Galindo *et al.* reported detection of 15 recombinant HERV-K (HML-2) transcripts in plasma from 7 HIV-1 infected patients and postulated that these sequences are the products of *in vivo* recombinations [44]. We also detected some recombinant sequences in all cell types, where several HERV-K (HML-2) loci are active, including PBMCs from healthy donors (Additional file 3). However, these sequences could be easily discriminated from genuine *in vivo* recombinants and could be explained as artifacts generated by *ex vivo* recombination during cDNA synthesis and amplification [73]. *In vivo* recombination would require co-packaging of two different HERV-K (HML-2) transcripts in an infectious HIV-1 particle followed by re-infection and reverse transcription of HERV-K (HML-2) RNA by presumably HIV machinery only then involving template switches. In our opinion, this would be a very rare event. In previous experiments using various retroviral vector systems, we have shown that HIV-1 derived systems co-package almost no HERVs in contrast to particles produced by MLV based packaging cell lines, which contain considerable amounts of HERV transcripts [24]. Therefore, we regard activation of HERV transcription by inhibition of restriction factors requiring re-infection unlikely and not to explain the substantial increase of HERV transcripts derived from different class I and II HERV groups.

Another possibility is the direct or indirect stimulation of HERV LTR promoters by trans-activating HIV-1 proteins binding to responsive HERV sequences or activating/inactivating cellular transcription factors. LTRs of HIV-1 and HERVs share many common features including binding sites for host cell factors required for formation of transcription initiation complexes and sequences involved in post-transcriptional regulation. For example, the HIV-1 Rev protein binds to a response element located within the HERV-K (HML-2) LTR [32]. Binding sites for transcription factors such as AP1, SP1, YY1 or glucocorticoid response elements present in the HIV-1 LTR have been identified in many HERV LTRs (reviewed in [74]). In particular, HERV-K (HML-2) LTRs have been extensively examined *in silico* and numerous potential transcription factor binding sites and response elements were detected, albeit in most cases the biological functionality has still to be verified [75].

Our experiments using various siRNAs directed against HIV-1 transcripts clearly show that HIV-1 gene products are responsible for up-regulation of at least four different HERV groups, HERV-T, HERV-E, ERV-9 and HERV-K (HML-10) in persistently infected LC5 cells. Recently, a recombinant HIV-1 Tat protein was reported to increase HERV-K (HML-2) transcription in Jurkat T-cells and primary lymphocytes [76]. The authors suggest an involvement of transcription factors NF-κB or NF-AT. Moreover, stimulation of LTRs from different HERV groups, in particular HERV-H, HERV-W and HERV-K (HML-4) by the HTLV-1 Tax protein, another retroviral transactivator with HIV-1 Tat-like functions, has been demonstrated by co-transfection of Jurkat T-cells with HERV-LTR-luciferase constructs and a Tax expressing vector [77]. HIV-1 encoded regulatory proteins like Tat and Nef are known to activate nuclear factors including NF-κB, NFAT and AP1 and thus stimulate HIV transcription [78,79].

To test the hypothesis of an involvement of cellular transcription factors in HIV-1 mediated HERV activation we stimulated Jurkat T-cells to induce a variety of transcription factors including NF-κB, NFAT and AP1 using agents known to activate HIV-1 expresssion [80]. As expected, many HERV groups show increased transcript levels and, moreover, the pattern of activated HERVs includes all HERV groups activated in *de novo* and in persistently HIV-1 infected cells. From this data we conclude that induction of transcription factor expression plays a crucial role in HIV-1 mediated HERV activation.

## Conclusions

In summary, our data demonstrate that productive HIV-1 infection is associated with alterations of HERV transcription patterns in human cells of different origin. Activation of HERV transcription is linked to levels of HIV-1 production in persistently infected cells and may be abrogated by HIV-1-specific siRNA indicating involvement of HIV-1 gene products in HERV activation. There are clear differences between HIV-1 activated HERVs in persistently and in *de novo* infected cells, suggesting differential modes of activation. Hence, identification of persistently HIV-1 infected cells by means of specifically expressed HERV transcripts/proteins as markers may be of diagnostic value. Furthermore, specific HERV proteins expressed by HIV-1 infected cells could serve as targets for adjuvant immunotherapies.

## Methods

### Cell culture

All cells were maintained in Biochrom VLE-RPMI 1640 with stable glutamine and 2.0 g/l NaHCO_3_ and 10% fetal calf serum (Seromed, Berlin, Germany). If applicable, 100 U/ml penicillin and 100 μg/ml streptomycin was added to the culture. Cells were cultured in an H_2_O-saturated atmosphere with 5% CO_2_ at 37°C. The human epithelia cell line LC5 is a HeLa derivative [50,81], and LC5-HIV cells are LC5 cells persistently infected with HIV-1IIIB/LAI [81]. LC5-RIC is a clonal LC5 cell line expressing a fluorescent HIV reporter (DsRed) [55]. The astrocytic cell line 85HG66 was established from a human brain tumor. TH4-7-5 is a persistently with HIV-1IIIB/LAI infected cell clone of 85HG66 cells [49]. The human T-lymphoma cell line KE37.1 and KE37.1-IIIB, the corresponding cell line persistently infected with HIV-1IIIB/LAI, are described in [49]. All cell lines were authenticated by the German Collection of Microorganisms and Cell Cultures (DSMZ). For *de novo* infection experiments LC5-RIC cells were infected with an HIV-1 patient isolate P-891 [54,55], cultured for three weeks and sorted for HIV positive cells. To study the time course of HIV-1 infection LC5-RIC cells were infected with HIV-1IIIB/LAI as described [54,55]. 3 days after virus exposure, cells were passaged for the first time and afterwards every 2–3 days when confluence reached 100%. Cells were cultured in the presence of selection antibiotics (Hygromycin B, Geneticin) to ensure ongoing overexpression of CD4 and the HIV-reporter construct [54,55].

### P24-antigen analysis

Quantification of p24 antigen in infected cells was performed using the Coulter HIV-1-p24-Antigen-Assay (Beckman Coulter) as described previously [82]. 5% Triton-X was added to the whole-cell extracts and to the supernatants, centrifuged at 13.000 rpm for 5 minutes at room temperature and diluted with PBS to a final concentration of 0.5% TritonX. The p24 ELISA was performed according to the manufacturer’s protocol.

### RNA preparation

Total RNA was extracted using a Qiagen RNeasy Mini Kit according to the manufacturers protocol. To remove genomic DNA contamination, all mRNA samples were treated with 1 U RNase-free DNase (Promega, Mannheim) per μg RNA. Subsequently, 25 ng of each mRNA preparation was tested by PCR with mixed oligo primers (MOP) [47] omitting the reverse transcription step. Only mRNA preparations negative for amplification products were used for subsequent reverse transcription and MOP multiplex PCR.

### cDNA synthesis and microarray experiments

Reverse transcription of mRNA was performed with 1μg total RNA using Superscript II (Roche Diagnostics, Mannheim) according to the manufacturers protocol. Three independent RNA isolations were used for microarray analysis. Amplification and labeling of the hybridization probes by MOP PCR, DNA microarray preparation, hybridization and post-processing of retrovirus-specific microarrays were performed as described previously [46,53]. Exclusively arrays showing reproducible hybridization patterns in triplicate subarrays were further evaluated. Hybridized microarrays were scanned using an Affymetrix Scanner GMS 418 (laser power settings, 100%; gain, 50%), and false color mapping was used for image visualization.

### Quantification of HERV and HIV-1 transcription by quantitative reverse transcriptase PCR (qRT-PCR)

For amplification of *pol* (RT) sequences, the following HERV subgroup-specific *pol* primers were used for S71pCRTK-1 (group HERV-T): reverse primer 5’-GTACCCCAGGTAGGAAACTCTGGG-3’, forward primer 5’-CCCCTACCCTTTTTGGGG-3’); E4-1 (group HERV-E): reverse primer 5’-GCTTTCTTTCTGATCCTAGGCTGTG-3’, forward primer 5’-CTTTGGGGAGGCGTTGGCTCGAGACC -3’; ERV-9 (group ERV9): reverse primer 5’-CCTCAACTGTTTTAATGTCTTAGGGCGAGG-3’, forward primer 5’-CCCTCATCTGTTTGGTCAGGCCC-3’); seq59 (group ERV9): reverse primer 5’-GTGCTGAGGGCCCTGGTTCCTCTGG-3’, forward primer 5’- CAGGCACAGGCCCAAGATCTAGTTC-3’; HERV-KC4 (group HERV-K (HML-10)): reverse primer 5’-GAATCTCTTCTAATTTGAACCTTTTGAGG-3’, forward primer 5’-CCCACAGTTTGTCAAACTTTTGTAGGC-3’; HIV-1: reverse primer 5’-GTTCATAACCCATCCAAAGGAATGGAGG-3’, forward primer 5’-CCAAAGTAGCATGACAAAAATC-3’. In general, HERV-specific primers for quantitative reverse transcriptase PCR (qRT-PCR) were designed in such a way that for each HERV one primer matched the capture probe sequences used in the corresponding microarray experiments whereas the second primer was located 100 to 150 base pairs upstream of the first primer [46]. For group HERV-K (HML-2), *gag*-specific primers were used for quantification (forward primer 5’-GGCCATCAGAGTCTAAACCACG-3’, reverse primer 5’-CTGACTTTCTGGGGGTGGCCG-3’) enabling a strict discrimination to other HERV-K groups such as HERV-K (HML-3). HIV-1 transcripts were amplified as described [83].

qRT-PCR was performed with the Roche LightCycler 1.5 System, using LightCycler FastStart DNA Master SYBR Green I-Kit and standard LightCycler protocol (Roche Diagnostics, Mannheim). Cycling conditions were a 10 minutes denaturation step at 95°C, followed by 40 cycles of 10 seconds at 95°C, 5 seconds at 60°C, and 10 seconds at 72°C. RNA-Polymerase II (RPII) transcripts were analyzed as internal standard, using primers given in [84]. To confirm that specific products were amplified, the PCRs were further analyzed by melting curve analysis and by agarose gel electrophoresis. ∆*C*_T_-values were calculated as follows: *C*_T_ (gene of interest) - *C*_T_ (house keeping gene). The relative transcription was calculated by the 2^-∆∆*C*^_T_ method [85]. Furthermore, extensive standardization of PCR reactions was initially performed through melting curve analysis of respective amplicons in order to minimize primer pair formation (data not shown). Absolute quantification of HIV proviral copies was performed as described previously [82].

### RNA interference

For down-regulation of HIV-1 gene expression, HIV-1-specific silencing RNAs (siRNA) [56] and non-silencing control RNAs (sin.s.) were synthesized (Qiagen). Transfections were carried out using RNAiFect transfection reagent (Qiagen) according to the manufacturer’s protocol. The day before transfection, 1 × 10^5^ target cells were seeded per well of a 12-well plate. 2 μg siRNA per well was used for each transfection. After 24 hours, medium was removed, cells were washed with PBS and new medium was added. Gene silencing was monitored by p24 analysis of the supernatant 72 hours after transfection. Total RNA of siRNA treated cells was extracted using a Qiagen RNeasy Mini Kit, reverse transcribed using the Superscript II Kit, and analyzed for HERV transcription using qRT-PCR.

### Amplification, cloning, and sequence analysis of HML-2 and HML-3 transcripts

For amplification of HML-2 transcripts, PCR was performed using 2 μl of undiluted cDNA. HERV-K (HML-2)-specific primers gag_plus (5’-GGCCATCAGAGTCTAAACCACG-3’) and gag_minus (5’-GCAGCCCTATTTCTTCGGACC-3’) were used to generate *gag* gene derived PCR products [58,86]. For amplification of the more divergent HERV-K (HML-3) *env* genes a multiplex PCR was carried out using the following primer combinations: HML3FOR (5’-TGTCGAGACTGATGCTGAGG-3’), HML3FOR_a (5’-TGTCAAGACTGACTCTGTGG-3’), HML3FOR_b (5’-TGTTGAGACTGATGCTGAGA-3’), HML3FOR_c (5’-TGTTGAGACTGACACTGAGA-3’) and HML3REV (5’- AATGATATGGCCCGCTGTAG-3’), HML3REV_a (5’- AATGATATGGGTTGTTGTAG-3’), HML3REV_b (5’- AATAATATGGCCTGCTGTAG-3’), HML3REV_c (5’-TATGATATGGCCCGCTGTAG-3’), HML3REV_d (5’- CATGATATGGCCCGCTCTAG-3’), HML3REV_e (5’-GATGATATGCCTGCTGTAG-3’), HML3REV_f (5’- CTAGAGCAGGCCATATCAGT-3’). The 50 μl PCR mix contained 1× Expand High Fidelity buffer with MgCl_2_, 0.2 μM deoxynucleotides, and 2.6 U expand High Fidelity enzyme mix (Roche Diagnostics, Mannheim). PCR conditions were as follows: initial denaturation at 94°C for 5 minutes; 40 cycles at 94°C for 1 minutes, annealing at 57°C for 45 seconds, and elongation at 72°C for 1 minutes, followed by a final elongation step at 72°C for 10 minutes. HML-2-specific *gag* PCR products were purified (NucleoSpin Extract II, Macherey-Nagel, Düren), cloned into the pGEM T-Easy vector (Promega) and used to transform TOP10F bacterial cells. Plasmid DNA was isolated from insert-containing colonies according to the manufacturer’s protocol (NucleoSpin Plasmid, Macherey-Nagel, Düren). Subsequently, cloned HML-2 cDNAs were analyzed by Sanger sequencing using an Applied Biosystems 3730 × Capillary Sequencer (Seq-IT, Kaiserslautern, Germany). We mapped individual cDNA sequences to their respective genomic loci in the March 2006 (NCBI36/hg18) version of the human genome sequence by using BLAT at the Human Genome Browser, as described previously [58,86].

### Stimulation of Jurkat T-cells with PMA/ionomycin and CD3/CD28 antibodies

2 × 10^6^ Jurkat T-cells were incubated with phorbol-12-myristate-13-acetate (PMA) and ionomycin or with antibodies against CD3 and CD28 in conditional complete RPMI medium [60]. For stimulation with PMA/ionomycin 200 ng/ml PMA and 300 ng/ml ionomycin were added directly to the medium (1 ml) and cells were incubated for 3 hours. For stimulation through CD3/CD28 antibody ligation the following antibodies were used to stimulate 2 × 10^6^ cells in 300 μl medium: 1 μg/ml anti-hCD3 (IgG2a), 5 μg/ml anti-hCD28 (IgG1), 2.5 μg/ml anti IgG1, and 2.5 μg/ml anti IgG2a. After 3 hours cells were placed on ice, washed once with cold PBS and RNA was isolated.

## Additional files

Additional file 1:
**Down-regulation of HIV-1 transcription by specific siRNAs.** (A) Binding sites of HIV-1-specific siRNAs used for down-regulation of HIV-1. (B) HIV-1 transcription in LC5-HIV cells treated with HIV-1 specific siRNAs. The Y-axis shows the x-fold relative HIV-1 transcript levels in LC5-HIV cells transfected with non-silencing siRNA (sin.s.) and HIV-1-specific siRNAs (sigag, sitat/rev, sinef, sienv) referred to uninfected control cells. Relative transcription was quantified according [85] and normalized to RPII transcript levels. The standard error for triplicate experiments is indicated in each bar. Additional file 2:
**Influence of overexpressed Risp proteins on HERV activity in persistently HIV-1 infected LC5 cells examined by a retrovirus-specific microarray False color mapping was used for image visualization.** The house keeping genes ubiquitin, glycerinaldehyde-3-phosphate-dehydrogenase (GAPDH) and hypoxanthine-guanine phosphoribosyltransferase (HPRT) served as a quality control and consequently as an internal standard. HIV-1 oligonucleotides are also spotted on the chip as a positive control for the infected cells and to demonstrate downregulation of HIV-1 transcription in cells overexpressing Risp. HERVs are grouped in class I, II and III elements. It should be noted that each positive spot on the microarray can represent multiple HERV proviruses of one multicopy HERV subgroup with sufficient sequence similarity that individual elements cannot be distinguished. HERV subgroups found up-regulated in all HIV-1 infected cell lines (see Figure 2) are displayed with red letters. Additional file 3:
**HERV-K (HML-2) transcription in PBMCs of six healthy individuals.**
Additional file 4:
**Provirus copy numbers of LC5-RIC cells infected with HIV1IIIB/LAI.** Relative transcription was quantified according to [85] and normalized to RPII transcript levels. Additional file 5:
**HERV-K (HML-3) transcription in** ***de novo*** **and persistently infected LC5 cells.**
Additional file 6:
**Relative transcription of IL-2 in PMA/ionomycin or CD3/CD28 stimulated Jurkat T-cells.** The Y-axis shows the x-fold relative transcription of IL-2 after PMA/ionomycin or CD3/CD28 stimulation referred to unstimulated cells. Relative transcription was quantified according to [85] and normalized to RPII transcript levels. Mean values and standard deviations are indicated for triplicate experiments performed with three independent RNA isolations.
