# Supplementary figures and images for: Modulation of human endogenous retrovirus (HERV) transcription during persistent and de novo HIV-1 infection
Source: Retrovirology. 2015 Mar 24;12:27. doi: 10.1186/s12977-015-0156-6 (PMC4375885; doi:10.1186/s12977-015-0156-6)

A

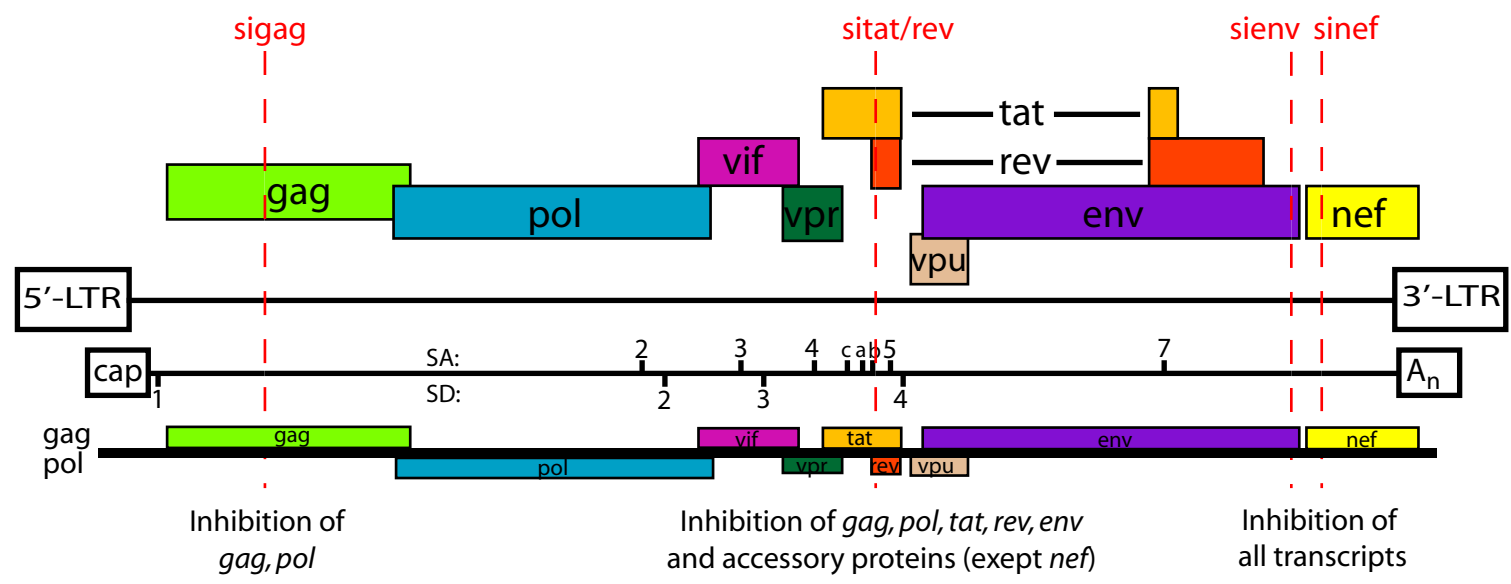

B

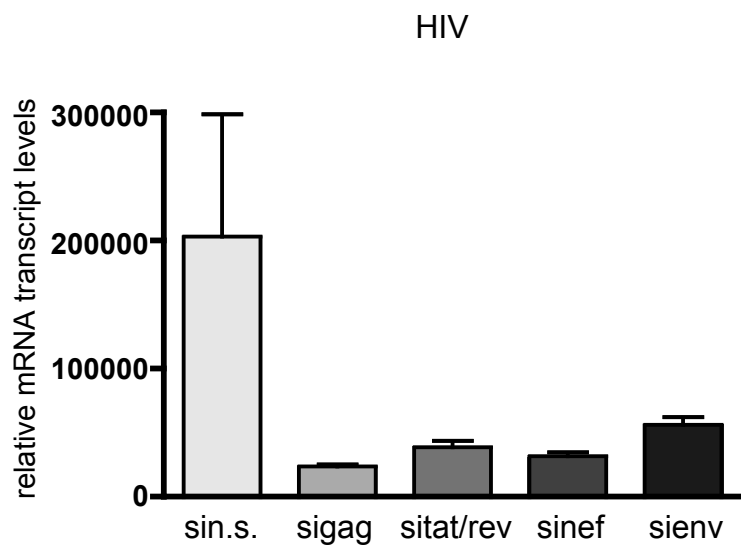

Supplement: Additional file 1: — Down-regulation of HIV-1 transcription by specific siRNAs. (A) Binding sites of HIV-1-specific siRNAs used for down-regulation of HIV-1. (B) HIV-1 transcription in LC5-HIV cells treated with HIV-1 specific siRNAs. The Y-axis shows the x-fold relative HIV-1 transcript levels in LC5-HIV cells transfected with non-silencing siRNA (sin.s.) and HIV-1-specific siRNAs (sigag, sitat/rev, sinef, sienv) referred to uninfected control cells. Relative transcription was quantified according [85] and normalized to RPII transcript levels. The standard error for triplicate experiments is indicated in each bar. [file 12977_2015_156_MOESM1_ESM.pdf]

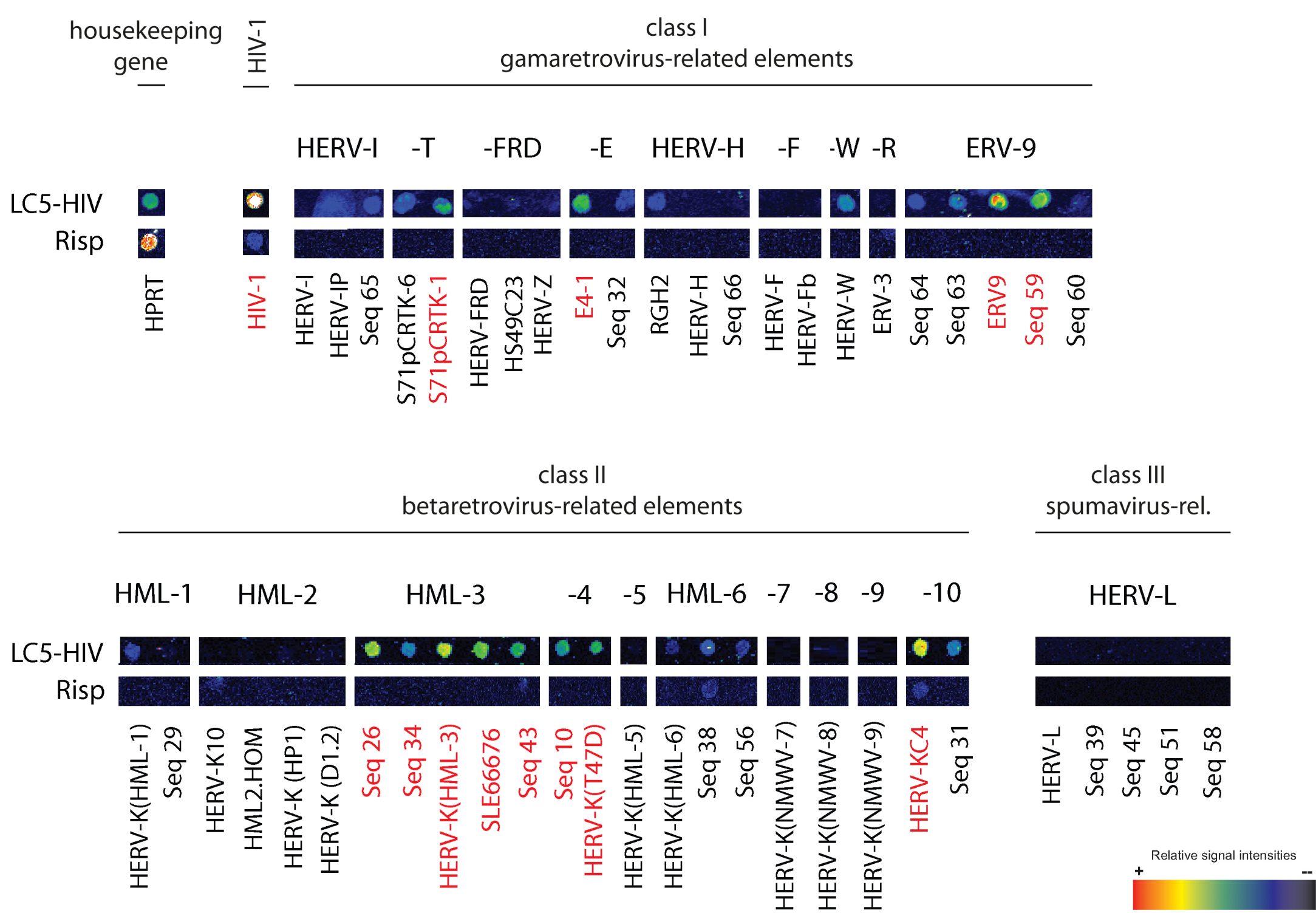

Supplement: Additional file 2: — Influence of overexpressed Risp proteins on HERV activity in persistently HIV-1 infected LC5 cells examined by a retrovirus-specific microarray False color mapping was used for image visualization. The house keeping genes ubiquitin, glycerinaldehyde-3-phosphate-dehydrogenase (GAPDH) and hypoxanthine-guanine phosphoribosyltransferase (HPRT) served as a quality control and consequently as an internal standard. HIV-1 oligonucleotides are also spotted on the chip as a positive control for the infected cells and to demonstrate downregulation of HIV-1 transcription in cells overexpressing Risp. HERVs are grouped in class I, II and III elements. It should be noted that each positive spot on the microarray can represent multiple HERV proviruses of one multicopy HERV subgroup with sufficient sequence similarity that individual elements cannot be distinguished. HERV subgroups found up-regulated in all HIV-1 infected cell lines (see Figure 2) are displayed with red letters. [file 12977_2015_156_MOESM2_ESM.pdf]

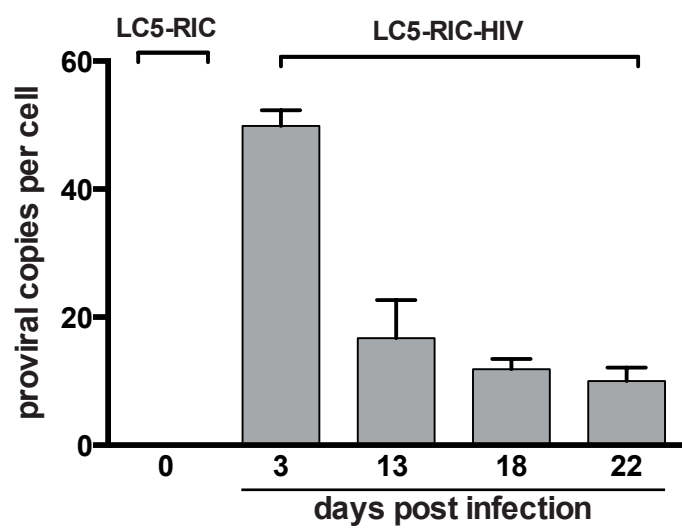

Supplement: Additional file 4: — Provirus copy numbers of LC5-RIC cells infected with HIV1IIIB/LAI. Relative transcription was quantified according to [85] and normalized to RPII transcript levels. [file 12977_2015_156_MOESM4_ESM.pdf]

## IL-2

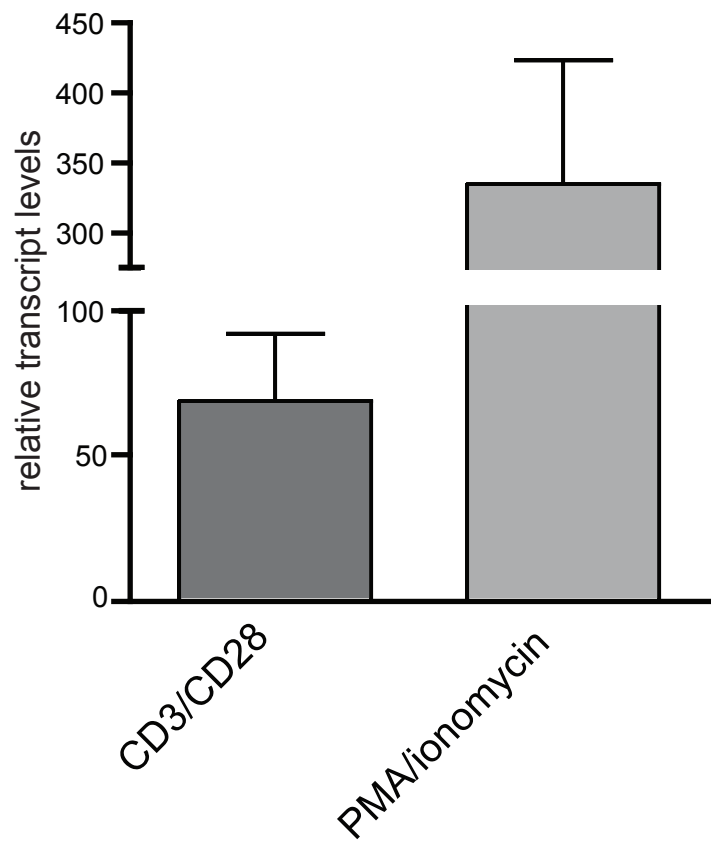

Supplement: Additional file 6: — Relative transcription of IL-2 in PMA/ionomycin or CD3/CD28 stimulated Jurkat T-cells. The Y-axis shows the x-fold relative transcription of IL-2 after PMA/ionomycin or CD3/CD28 stimulation referred to unstimulated cells. Relative transcription was quantified according to [85] and normalized to RPII transcript levels. Mean values and standard deviations are indicated for triplicate experiments performed with three independent RNA isolations. [file 12977_2015_156_MOESM6_ESM.pdf]
