# Supplementary material for: Modulation of human endogenous retrovirus (HERV) transcription during persistent and de novo HIV-1 infection
Source: Retrovirology. 2015 Mar 24;12:27. doi: 10.1186/s12977-015-0156-6 (PMC4375885; doi:10.1186/s12977-015-0156-6)
Supplement: Additional file 3: — HERV-K (HML-2) transcription in PBMCs of six healthy individuals. [file 12977_2015_156_MOESM3_ESM.pdf]

HERV-K(HML-2) activity in PBMCs of six healthy individuals

Provirus[a]	Chromosome band	Number of clones (% cloning frequency)					
		1	2	3	4	5	6
ERVK-7[b] (K102, K50a, c1_B)	1q22	8 (36.4)	15 (75.0)	5 (19.2)	4 (23.5)	9 (50.0)	21 (87.5)
ERVK-18 (K110, K18, c1_C)	1q23.3	9 (40.9)	2 (10.0)	15 (57.7)	10 (58.8)	4 (22.2)	-
ERVK-5 (K(II))	3q12.3	-	-	2 (7.7)	-	1 (5.6)	1 (4.2)
ERVK-3[b,c] (K106, K68, c3_B)	3q13.2	-	-	1 (3.8)	-	-	1 (4.2)
ERVK-11[b] (K117, K50b)	3q27.2	1 (4.5)	-	-	-	-	-
ERVK-10[b] (K107, K10, c5)	5q33.3	1 (4.5)	-	-	-	-	-
ERVK-8[b] (K115)	8p23.1	-	1 (5.0)	-	2 (11.8)	-	-
ERVK-17[b] (c10_B)	10q24.2	-	-	1 (3.8)	-	-	-
K(OLD-AC004127)	11q12.3	-	-	-	-	1 (5.6)	-
ERVK-20 (K37, c11_B)	11q23.3	-	-	1 (3.8)	-	-	-
ERVK-19[b] (c19)	19q11	-	-	-	-	-	1 (4.2)
K(OLD-AC012309)	19q13.12	3 (13.6)	1 (5.0)	1 (3.8)	1 (5.9)	3 (16.7)	
Total number of clones		22 (100.0)	20 (100.0)	26 (100.0)	17 (100.0)	18 (100.0)	24 (100.0)

[a] Designation according to [87], aliases are in parentheses.
[b] Human specific HERV-K(HML-2) proviruses [89].
[c] Polymorphic in the human genome [89].
