# Supplementary material for: Modulation of human endogenous retrovirus (HERV) transcription during persistent and de novo HIV-1 infection
Source: Retrovirology. 2015 Mar 24;12:27. doi: 10.1186/s12977-015-0156-6 (PMC4375885; doi:10.1186/s12977-015-0156-6)
Supplement: Additional file 5: — HERV-K (HML-3) transcription in de novo and persistently infected LC5 cells. [file 12977_2015_156_MOESM5_ESM.pdf]

HERV-K(HML-3) transcription in *de novo* and persistently infected LC5 cells

Provirus[a]	Chromosome band	Location of amplicon in genome[b]	Number of clones (% cloning frequency)			
			LC5-RIC	LC5-RIC-HIV[c]	LC5	LC5-HIV[d]
ERVK9-1 (K102B)	1q21.2	148897257 - 148897929	28 (57.1)	23 (51.1)	13 (28.9)	29 (46,7)
ERVK9-2 (K22C)	2p24.1	19990171 - 19990837	-	2 (4.4)	-	2 (3.2)
K105B[e]	3q21.3	131107276 - 131107940	8 (16.3)	13 (28.9)	17 (37.7)	19 (30.6)
ERVK9-3 (K26C)	3q27.2	186214162 - 186214832	2 (4.1)	-	-	3 (4.8)
ERVK9-4 (K83B)	5p13.2	34818528 - 34819198	1 (2.0)	1 (2.2)	1 (2.2)	3 (4.8)
ERVK9-5 (K104B)	5p15.33	1631842 - 1632506	4 (8.2)	4 (8.9)	-	-
ERVK9-6 (K58B)	6q21	111349419 - 111350092	-	-	1 (2.2)	-
ERVK9-7 (K103B)	7p14.2	35917184 - 35917857	3 (6.1)	2 (4.4)	3 (6.7)	2 (3.2)
ERVK9-8 (K49B)	8q11.23	55150562 - 55151221	-	-	1 (2.2)	-
ERVK9-9 (K18B)	17p13.2	6720109 - 6720772	-	-	1 (2.2)	-
ERVK9-10 (K114B)	17q21.32	44603909 - 44604585	-	-	5 (11.1)	2 (3.2)
ERVK9-11 (K79B)	19q13.2	44118558 - 44119192	3 (6.1)	-	3 (6.7)	2 (3.2)
	Total number of clones		49 (100)	45 (100)	45 (100)	62 (100)

[a] Designation according to [87], aliases are in parentheses.
[b] According to the March 2006 assembly (NCBI36/hg18) of the human genome sequence at the Human Genome Browser [88].
[c] *de novo* infected LC5-RIC cells.
[d] persistently infected LC5 cells.
[e] The provirus sequence is transcribed as part of lncRNA TMCC1-AS1.
